# Characterization of the Medium- and Long-Chain *n*-Alkanes Degrading *Pseudomonas aeruginosa* Strain SJTD-1 and Its Alkane Hydroxylase Genes

**DOI:** 10.1371/journal.pone.0105506

**Published:** 2014-08-28

**Authors:** Huan Liu, Jing Xu, Rubing Liang, Jianhua Liu

**Affiliations:** State Key Laboratory of Microbial Metabolism, and School of Life Sciences & Biotechnology, Shanghai Jiaotong University, Shanghai, China; Loyola University Medical Center, United States of America

## Abstract

A gram-negative aliphatic hydrocarbon-degrading bacterium SJTD-1 isolated from oil-contaminated soil was identified as *Pseudomonas aeruginosa* by comparative analyses of the 16S rRNA sequence, phenotype, and physiological features. SJTD-1 could efficiently mineralize medium- and long-chain *n-*alkanes (C_12_-C_30_) as its sole carbon source within seven days, showing the most optimal growth on *n*-hexadecane, followed by *n*-octadecane, and *n*-eicosane. In 36 h, 500 mg/L of tetradecane, hexadecane, and octadecane were transformed completely; and 2 g/L *n*-hexadecane was degraded to undetectable levels within 72 h. Two putative alkane-degrading genes (gene 3623 and gene 4712) were characterized and our results indicated that their gene products were rate-limiting enzymes involved in the synergetic catabolism of C_12_–C_16_ alkanes. On the basis of bioinformatics and transcriptional analysis, two P450 monooxygenases, along with a putative AlmA-like oxygenase, were examined. Genetically defective mutants lacking the characteristic alkane hydroxylase failed to degrade *n*-octadecane, thereby suggesting a different catalytic mechanism for the microbial transformation of alkanes with chain lengths over C_18_.

## Introduction

Oil pollution poses a severe threat to ecosystems, because of its refractory resistance to environmental degradation. Many remediation technologies have been used for the removal of contaminating oil residues [1]. Bioremediation is considered to be a safe, efficient, and economically viable alternative to physicochemical methods for the elimination of oil contaminants. In the past 30 years, several research groups around the world have tried to build robust biocatalysts for the efficient removal of contaminating oil residues. Although these attempts have not succeeded so far, the constant search for an effective biocatalyst has resulted in the discovery of a large number of microorganisms that could be used for bioremediation [2], [3].

Alkanes are the most abundant hydrocarbons (with an estimated abundance of 20–50%) in crude oil [4]. As alkanes are non-polar molecules with very low chemical activity, their utilization by microorganisms faces significant challenges, owing to known factors, such as low water solubility, high degree of accumulation in cell membranes, and higher activation energies [5], [6]. Nonetheless, at least sixty genera of aerobic bacteria, and five genera of anaerobic bacteria [7], such as *Pseudomonas* [8], *Acinetobacter* [9], *Rhodococcus* [10] and *Dietzia* [11], have been reported to possess the ability to degrade aliphatic hydrocarbons. Among them, *Pseudomonas* was found both in soil as well as in aqueous environments [12]. Several *Pseudomonas* strains are known to utilize aliphatic hydrocarbons as its sole carbon sources [13]–[15]. *P. aeruginosa* RR1 and *P. fluorescens* CHA0 degrade *n-*alkanes ranging from C_12_ to C_34_ [16], [17]; and *P. aeruginosa* DQ8 can grow in the presence of *n*-tetrodecane, *n*-docosane, *n*-triacontane, and *n*-tetrocontane [8].

Although many strains have been reported to utilize hydrocarbons, most of them can only use a narrow range of substrates. For example, *Bacillus stearothermophilus* can only grow in media containing hydrocarbons of chain length C_15_ to C_17_ [18], whereas *A. borkumensis* AP1, SK2, and SK7 can only utilize alkanes ranging from C_6_ to C_16_ [19]. Indeed, very few strains such as *Acinetobacter baylyi* ADP1 and *Thermus sp*. C2 can degrade a wide range of hydrocarbons. However, their genetic characteristics remain elusive and not much is known about the mechanisms through which these microorganisms break down long chain alkanes present in refractory oil residues [20], [21].

In this study, a *P. aeruginosa* strain SJTD-1 was isolated from oil-contaminated soil; its hydrocarbon utilization capability and *n*-alkane breakdown efficiency were investigated. Based on its whole-genome DNA sequence [22], several alkane hydroxylases were characterized. These included two AlkB monooxygenases, two P450 monooxygenases, and one AlmA-like monooxygenase. Varying transcriptional expression levels of these genes induced by C_12_–C_24_ alkanes indicated the presence of a complex hydrocarbon breakdown mechanism in *P. aeruginosa* SJTD −1.

## Materials and Methods

### Chemicals and media

*n*-Decane (>99% pure) was purchased from Alfa Aesar Organic Co., Inc (Tianjing); *n*-dodecane, *n*-tetradecane, *n*-hexadecane, and *n*-octadecane (all >99% pure) from Sangon (Shanghai, China); and *n*-pentadecane, *n*-eicosane, *n*-docosane, *n*-tetracosane, *n*-triacontane, and *n*-hexane (of HPLC gradient grade) from Sigma-Aldrich (St. Louis, MO, USA). All other reagents used in this study were of analytical reagent grade.

Luria-Bertani (LB) medium (tryptone 10.0 g, yeast extract 5.0 g, NaCl 10 g/L) and basal salt medium (BSM) (K_2_HPO_4_ 3.815 g, KH_2_PO_4_ 0.5 g, (NH_4_)_2_HPO_4_ 0.825 g, KNO_3_ 1.2625 g, Na_2_SO_4_ 0.2 g, CaCl_2_ 0.02 g, FeCl_3_ 0.002 g, and MgCl_2_ 0.02 g/L) were used in this study. To examine the utilization of alkanes by strain SJTD-1, both liquid and solid alkanes, maintained at room temperature, were first dissolved in *n*-hexane to form 500 mg/mL alkane-hexane solutions. These solutions were then added into the BSM medium to attain various concentrations. The *n*-hexane was neither toxic to the strain, nor was it utilized by the strain.

### Strain Isolation

Samples used for bacterial enrichment were collected from the oil-contaminated soil in the Daqing Oil Field of China (no specific permissions were required and this work did not involve any endangered or protected species). Approximately 5 g of the soil sample was inoculated into a 250 mL flask with 100 mL BSM liquid medium containing 2 g/L hexadecane, and was shaken at 180 rpm for seven days, at 37°C. A 5 mL culture was then inoculated into 100 mL of fresh BSM liquid medium with *n*-hexadecane, and cultured as above. Enrichment cultures after rounds of enrichment were diluted and plated onto BSM agar plates pre-coated with hexadecane. Bacterial colonies grown on plates with different morphology were tested for their *n*-alkane utilizing capabilities. One strain, which exhibited a fast growth rate was purified and designated as SJTD-1.

### 16S rRNA analysis and phylogenic tree construction

The newly isolated strain SJTD-1 was identified based on its morphological, physiological, and biochemical properties listed in Bergey’s Manual of Determinative Bacteriology [23]. After its genomic DNA was extracted with standard molecular biology techniques [24], the 16S rRNA gene of strain SJTD-1 was amplified using Bacterial 16S rDNA Kit (TaKaRa Biotechnology Co., Ltd. Dalian, China). The PCR program is first denaturized at 94 °C for 5 min, then denaturized at 94 °C for 1 min, annealed at 55 °C for 1 min and elongated at 72 °C for 1.5 min; Repeated this process for 30 times, and then further elongated at 72°C for another 5 min. After the PCR reaction, the fragments were sequenced using the primer pairs 16S-seq-F/R (Table S1), and this 16S rRNA sequence was deposited into GenBank (Accession No. JQ951926.1). A phylogenic tree based on the 16S rRNA sequences of the SJTD-1 strain and some other strains was analyzed with MEGA 5.0, using the Neighbor Joining method with 1,000 replications. The genetic distances were calculated with the Kimura two-parameter distance model.

### Growth rate of SJTD-1 in pure *n-*alkanes

The cell growth rate of strain SJTD-1 in *n-*alkanes was determined by the Automatic Growth Curve Analyzer (BioScreen Testing Service, Inc., CA, USA). Briefly, a single colony of strain SJTD-1 was inoculat**e**d into a 100 mL baffled flask containing 10 mL LB broth, and cultured on a rotary shaker overnight at 37°C. The cells were then harvested by centrifugation at 8,000 rpm for 5 min and washed thrice with sterilized water to exclude the residual organic compounds. The cells were then re-suspended in the BSM medium to OD_600_≈2.0, to form the inoculum. Subsequently, 10 µL of the cell pellets were inoculated into the 500 µL well of a 10×10 multi-well plate, which contained 190 µL of the BSM medium amended with pure *n-*alkanes (C_10_–C_24_) of different concentrations. The initial cell concentration in each well at OD_600_ was 0.1. All *n-*alkanes were prepared as *n*-hexane solutions. Wells containing cells without *n-*alkanes and those containing *n-*alkanes without inoculum were used as blanks. The 10×10 multi-well plates were loaded onto the Automatic Growth Curve Analyzer and incubated at 30°C with constant shaking (180 rpm) for seven days. The cell densities were determined by detecting the OD_600_ every hour. All experiments were repeated thrice, and the results shown were the average values of three replicates, along with the standard errors.

### Degradation analysis of SJTD-1 in the case of pure *n-*alkanes

The degradation efficiencies of strain SJTD-1 for *n-*alkanes of different carbon lengths (C_14_–C_16_) were determined according to the loss of substrate from each liquid culture. A SJTD-1 cell inoculum was prepared as described above. The cells were distributed in flasks containing 100 mL BSM medium, amended with 500 mg/L of pure C_10_–C_24_ *n-*alkanes (which was the sole carbon source), and cultured for seven days at 30°C in the shaker. The initial OD_600_ was 0.1. Flasks without cell inoculum were used as blanks to assess the abiotic loss. The cultures were taken out at different time points, to estimate the cell concentrations and the alkane residues. For the residual analysis of alkanes, 3 mL cell cultures were collected into 10 mL-glass centrifuge tubes and extracted with 1 mL *n*-hexane for the first time. The mixtures were shaken vigorously, centrifuged at 5,000 rpm for 10 min. Then, the organic layer was collected and the aqueous layer was extracted twice, with 1 mL *n*-hexane each time. The organic extracts were pooled together to a final volume of 3 mL, and dried with anhydrous sodium sulfate. For each sample, *n*-pentadecane (100 mg/L) was added before the extraction and used as an internal standard. All extractions were performed in triplicate, and the results were expressed as average values with standard errors.

The degradation efficiencies of strain SJTD-1 for *n*-hexadecane of various concentrations (250, 500, 1,000, 2,000 mg/L) were also analyzed as above. After strain SJTD-1 was cultured for one to seven days, the residual *n*-hexadecane at different time points were extracted and determined with three replicates.

### Analytical methods of *n-*alkanes concentration

The concentrations of *n-*alkanes were determined with the gas chromatography-mass spectrometry (GC-MS) technique, using a GC/MS system (7890A GC/5975C MS, Agilent, USA) equipped with a fused DB-5 MS capillary column (0.25 mm×30 m×0.25 µm). The GC program was listed as below. Helium was used as the carrier gas with a constant flow rate of 1.0 mL/min. The split ratio was 10∶1, and the temperatures of the injector and the connector were 270°C and 280°C, respectively. The temperatures of the ion source and the quadrupole were 230°C and 150°C, respectively. The column oven temperature was kept at 150°C for 2 min, and then raised to 200°C at a rate of 5°C/min, followed by an increase to 290°C, at a rate of 30°C/min, and at an isotherm of 290°C. The ionization mode was set as EI+, 70 ev, and the voltage of the detector was 1,388 V. The GC-MS spectra were analyzed with the ChemStation software, and the relative abundance of different hydrocarbon residues was calculated by the ratio of the peak area of each hydrocarbon to the peak area of *n*-pentadecane in the GC chromatograph. The residue ratio of *n-*alkanes was calculated with the equation *R* = [*S*]/[*I*], where *R*, [*S*], and [*I*] represent the alkane residue ratio, the residual *n*-alkane concentrations in the samples, and the concentration before inoculation, respectively. The results were expressed as mean values with standard deviation. The cell-free controls were incubated and analyzed in the same manner.

### Deletion of the putative alkane-degradation genes from strain SJTD-1

Based on the whole-genome DNA sequence of strain SJTD-1 [22], two putative *alkB* genes, gene 3623 and gene 4712, showed great similarities to the reported *alkB2* and *alkB1* genes, respectively. To determine the function of *alkB1* and *alkB2*, the knockout mutants of the single gene and two genes were constructed using the pRKaraRed homologous recombination system [25]. Briefly, plasmid pRKaraRed was first electro-transformed into strain SJTD-1 to generate the SJTD-1/pRKaraRed competent cells. The in-frame scarless knockout mutant of the single gene was generated by two-step homologous recombination. First, the *sacB-bla* cassettes were amplified from plasmid pEX18Ap [26] with primer pairs F1 and R1, and then electro-transformed into the SJTD-1/pRKaraRed competent cells. The transformants were screened on LB plates supplemented with 500 µg/mL carbenicillin and 50 µg/mL tetracyclin. Next, the *sacB-bla* removal cassettes amplified from the genomic DNA of the first-step colonies (Suc^S^Carb^R^) using the primer pairs F2 and R2, were electro-transformed into the competent cells of the first step to perform the second recombination. The transformants were spread on LB plates with 10% sucrose and 50 µg/mL tetracycline. The transformants were further selected in parallel on LB plates with 10% sucrose and 50 µg/mL tetracycline, and on LB plates with 500 µg/mL carbenicillin and 50 µg/mL tetracycline. A positive genotype (Suc^R^Carb^S^) was verified by PCR detection with the test primers and DNA sequencing was performed by Invitrogen. The deletion of multiple genes was achieved by repeating this two-step knockout method. Two *alk*B genes in strain SJTD-1 were knocked out separately or simultaneously, and the mutant strains were named as Mut*_alkB1_*, Mut*_alkB2_*, and Mut*_alkB1&2_*, respectively. Similarly, two putative P450 genes (gene 5482 and 4609), and one AlmA-like monooxygenase gene (gene 3206) were also deleted and the mutant strains were Mut*_P450-1_*, Mut*_P450-2_*, and Mut*_AlmA_*, respectively. All the mutants are listed in Table 1; all the primers for homologous recombination and PCR detection are listed in Table S1. The sequences of the five alkane hydroxylase genes are shown in Text S1.

**Table 1:** Strains, plasmids and putative genes studied in the work.

Name	Description	Source/reference
**Strains**		
*P. aeruginosa* SJTD-1	Host strain isolated from oil-contaminated soil, Wild Type	This study
Mut*_alkB1_*/Mut*_alkB2_*/Mut*_alkB1&2_*	AlkB-deleted mutants of *P. aeruginosa* SJTD-1	This study
Mut*_P450-1_*/Mut*_P450-2_*/Mut*_P450-1&2_*	P450-deleted mutants of *P. aeruginosa* SJTD-1	This study
Mut*_almA_*	AlmA-deleted mutants of *P. aeruginosa* SJTD-1	This study
**Plasmids**		
pRKaraRed	Broad-host-range, λ-Red proteins expression vector, Tet^R^	[25]
**Genes** *		
4712	alkane-1 monooxygenase, AlkB1	This study
3623	alkane-1 monooxygenase, AlkB2	This study
5482	putative cytochrome P450 hydroxylase, P450–1	This study
4609	putative cytochrome P450 hydroxylase, P450–2	This study
3206	monooxygenase, flavin-binding family, AlmA-like protein	This study

*Gene numbers presented in RAST server.

To study the roles of these alkane hydroxylases in *n*-alkane utilization, the growth curves of strain SJTD-1 and mutants (with different *n-*alkanes as the sole carbon sources) were determined by the Automatic Growth Curve Analyzer as described above. Experiments were replicated thrice and the results obtained were the averages values with standard errors.

### Quantitative reverse transcription-PCR (Q-RT-PCR)

To study the transcription profiles of the five alkane hydroxylases in different conditions, the wild type and mutant SJTD-1 were cultured in the BSM medium with sodium acetate and various alkanes (C_12_–C_24_) to the mid-exponential phase. The alkanes were the sole carbon sources. Total RNA was extracted using the FastRNAPro Blue Kit for microbes (MP Biomedicals, Santa Ana, CA) and the RNA extraction was performed according to the manufacturer’s instructions. The RNA yield was estimated using a Nanodrop UV spectrometer (Thermo Scientific, Wilmington, DE, USA). The reverse transcription was achieved using the PrimeScript Reverse Transcriptase Kit (TaKaRa, Dalian, China). Approximately 1 µg RNA and 20 ng random primers were used. The Q-RT-PCR was performed using the IQSYBR Green Super-mix (Bio-Rad Laboratories, Hercules, CA) and gene-specific primers (RT-F and RT-R, Table S1) in the IQTM 5 Multicolor Real-time PCR Detection System (Bio-Rad, CA, USA). The Q-RT-PCR procedure carried out was as follows: 15 min of pre-denaturing, 40 cycles of 95°C for 10 s; 60°C for 30 s; and 72°C for 30 s; followed by a melting curve stage from 60°C to 95°C. The cDNA amplification efficiency of the samples, internal standards (16S rRNA), and calibrators (samples without induction by alkanes) were equivalently modulated, and the relative fold change in mRNA quantity was calculated using the DDCt method [27]. At least three independent Q-RT-PCR experiments were conducted for each RNA sample. The change in the transcriptional levels of strains grown with alkanes was calculated by comparing with the transcriptional level of the strains grown in sodium acetate [17].

## Results

### Isolation and identification of the oil-degrading strain SJTD-1

**Figure 1:**
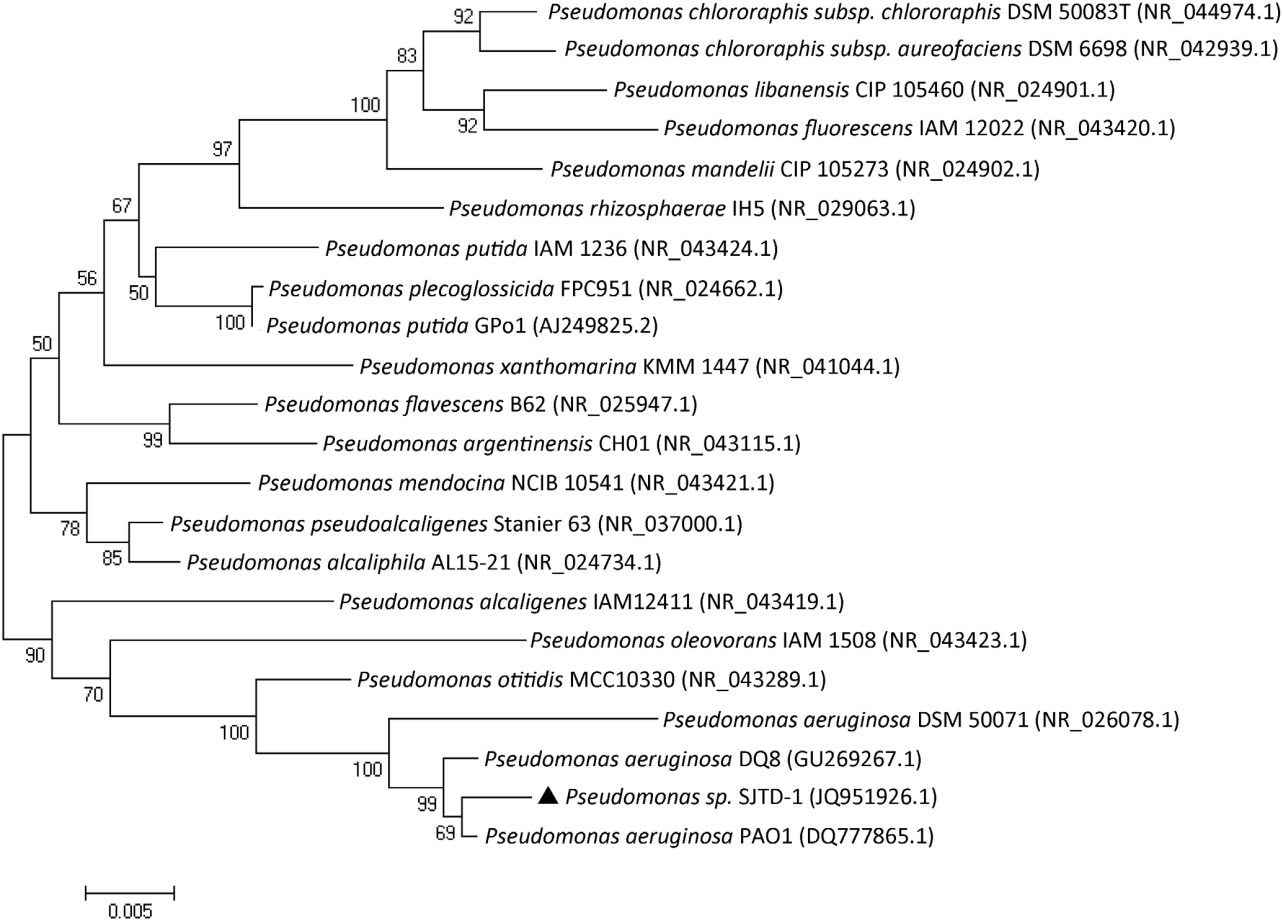
Phylogenetic tree based on 16S rDNA gene sequences indic*a*ting the SJTD-1 *position.* *Th*e Kimura two-parameter distance model was used, and bootstrap analysis was performed with 1,000 repetitions using MEGA 5.0.

Strain SJTD-1 isolated from the enrichment culture of oil-contaminated soil is a rod-shaped, gram-negative bacterium. Its growth pH ranged from 4.0 to 10.0, and optimal growth occurred at pH 7.0. After growing on LB agar at 37°C for 24 h, the cells formed green, round, moist and glossy colonies, approximately 1.0 mm in diameter. Strain SJTD-1 could utilize *n-*alkanes, from *n*-dodecane (C_12_) to *n*-triacontane (C_30_), as its (sole) carbon sources. Although SJTD-1 could not grow in the presence of *n-*decane or other shorter length *n-*alkanes, its growth was not inhibited by the short-chain *n-*alkanes like *n-*hexane. The 16S rRNA gene sequence of SJTD-1 (Genebank Accession No. JQ951926.1) was 99% identical to that of *P. aeruginosa* PAO1 (Genebank Accession No. DQ777865.1), and the corresponding phylogenetic tree analysis supported a strong relationship between SJTD-1 and members of *Pseudomonas sp*. (Fig. 1). Therefore, SJTD-1 was classified as a *P. aeruginosa* strain.

### Growth curve of strain SJTD-1 and its *n*-alkane degradation efficiency

**Figure 2:**
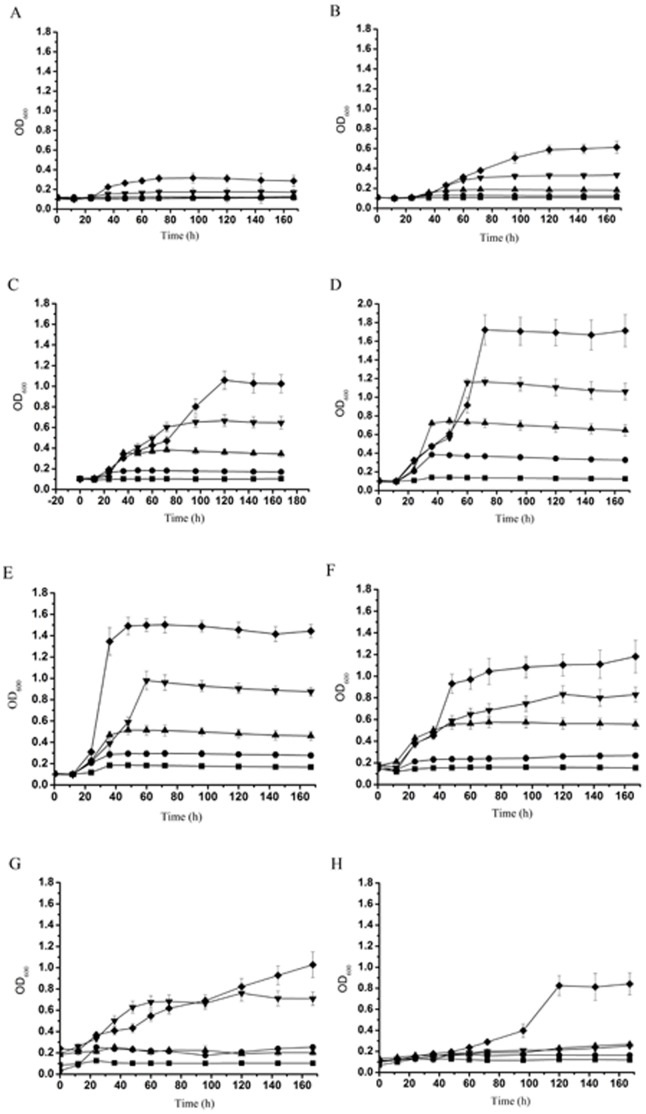
Growth curves of *P. aeruginosa* SJTD-1 in *n-*alkanes. Strain SJTD-1 was cultured in BSM supplemented with different concentrations (▪ 100 mg/L, • 250 mg/L, ▴ 500 mg/L, ▾ 1,000 mg/L, and ♦ 2,000 mg/L) of *n-*alkanes: *n*-decane (A), *n*-dodecane (B), *n*-tetradecane (C), *n*-hexadecane (D), *n*-octadecane (E), *n*-eicosane (F), *n*-docosane (G), and *n*-tetracosane (H) for seven days at 30°C. The data are expressed as mean values and the standard deviations are shown.

The SJTD-1-mediated breakdown and utilization of *n-*alkanes was studied by monitoring its cell growth for seven days in a BSM medium containing a variety of *n-*alkanes (C_10_–C_24_) at various concentrations (100 mg/L to 2 g/L) (Fig. 2). Cells started multiplying immediately after incubation and reached the exponential growth phase between day 1 and day 3. Of all the *n-*alkanes, *n-*hexadecane caused the highest amount of cell growth, followed by *n-*octadecane and *n-*eicosane, thus suggesting that *n-*hexadecane was probably the best available carbon source for strain SJTD-1 in this set of experiments. At higher concentrations of *n-*alkanes, strain SJTD-1 accumulated more biomass (as reflected by OD_600_), and also required more time to reach the stationary phase. The stationary phase was attained after 36 h when 500 mg/L of *n-*tetradecane, *n-*hexadecane, *n-*octadecane, and *n-*eicosane were used. At 1 g/L of *n-*dodecane and *n-*docosane, the stationary phase was reached after 60 h and 36 h, respectively. When 2 g/L *n-*tetracosane was used, the stationary phase was attained after nearly 100 h (Fig. 2).

**Figure 3:**
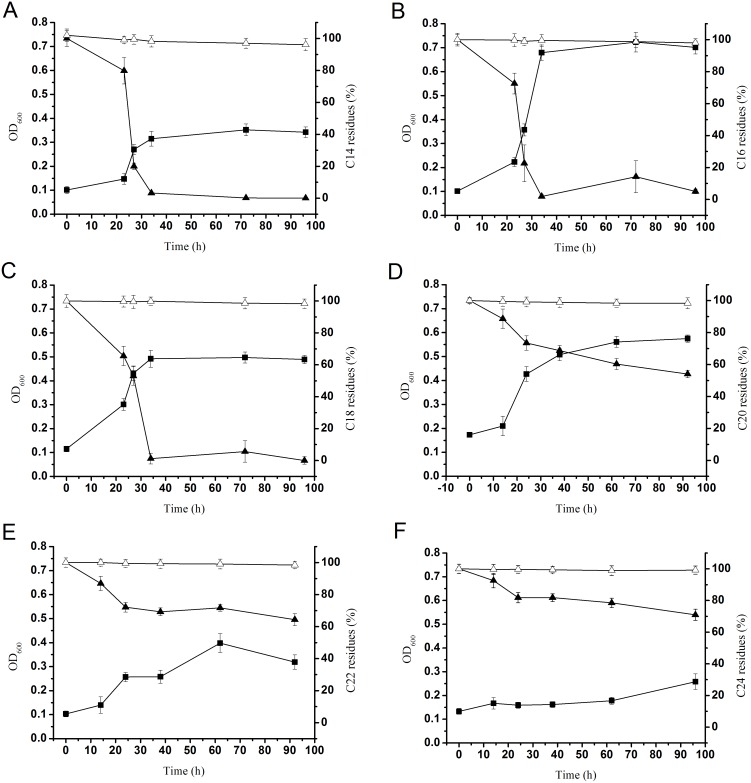
Growth of strain SJTD-1 in different *n-*alkanes (500 mg/L) and its consumption of *n-*alkanes. ▪ indicated as OD_600_ in the left vertical axis; ▴expressed as *n*-alkane residues detected by GC-MS with respect to abiotic controls in the right vertical axis); Δ indicated as the *n*-alkane residues of the abiotic controls. A, B, C, D, E, and F represented the *n-*alkanes C_14_, C_16_, C_18_, C_20_, C_22_, and C_24_, respectively. Standard errors were calculated from three independent determinations.

We also determined the *n*-alkane degradation efficiency of strain SJTD-1 with GC-MS. The corresponding time courses are illustrated in Fig. 3. GC-MS results showed that substrate consumption was closely followed by biomass increase (the loss due to vaporization and extraction was excluded by running two blank controls). As shown in Fig. 3A to 3C, more than 95% of 500 mg/L *n*-tetrodecane, *n*-hexadecane, and *n*-octadecane were degraded within 36 h (from 500 mg/L to about 15 mg/L, 5 mg/L, and 5 mg/L, respectively). Complete degradation occurred in less than 48 h, and the biomass at OD_600_ of *n*-tetrodecane, *n*-hexadecane, and *n*-octadecane increased to 0.35, 0.72, and 0.50, respectively. It was obvious that the degradation rates of strain SJTD-1 for various lengths of *n-*alkanes were different. Strain SJTD-1 could degrade *n*-eicosane, *n*-docosane, and *n*-tetracosane completely until the end of the experiments (96 h or 160 h). Only about 50% of the *n*-eicosane was degraded in 96 h. Although a rapid decline to about 70% residues was observed in the first 24 h, the degradation rate decreased in subsequent hours and the net biomass accumulation corresponded to an OD_600_≈0.58 (Fig. 3D). Similarly, the utilization efficiencies for *n*-docosane and *n*-tetracosane were about 35% and 25%, respectively, and their respective biomass reached an OD_600_ of approximately 0.39 and 0.26, respectively (Fig. 3E and 3F). All these indicated that strain SJTD-1 degraded the medium-chain *n-*alkanes more rapidly than the long-chain *n-*alkanes, probably due to the much higher hydrophobicity of the latter [17], [28], [29].

**Figure 4:**
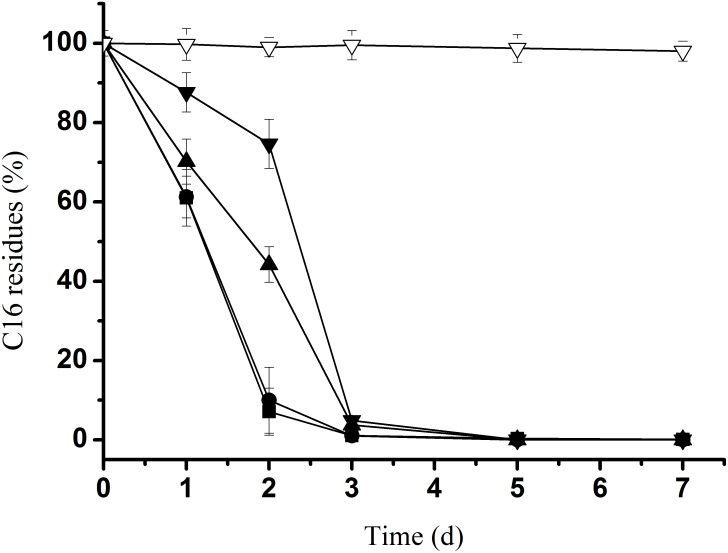
Quantitative estimation of the SJTD-1-mediated degradation of *n*-hexadecane. The strain was cultured in BSM supplemented with 250 mg/L (▪), 500 mg/L (•), 1,000 mg/L (▴), and 2,000 mg/L (▾) of *n*-hexadecane at 30°C for seven days; Δ indicated as the *n*-alkane residues of the abiotic controls. Standard errors were calculated from three independent determinations.

Moreover, to find the highest concentration of *n-*alkanes that the SJTD-1 strain could tolerate and utilize for growth, we analyzed its degradation efficiency for *n*-hexadecane (concentration ranging from 250 mg/L to 2.0 g/L) because *n*-hexadecane was possibly its greatest carbon source. As shown in Fig. 4, all the *n*-hexadecane could be completely degraded in three days. Additionally, 250 mg/L and 500 mg/L of *n*-hexadecane were totally degraded within 36 h; as for 1.0 g/L and 2.0 g/L *n*-hexadecane, approximately one more day was needed for a complete degradation. Therefore, we concluded that SJTD-1 could tolerate and completely biotransform 2.0 g/L *n*-hexadecane into a relatively large amount of biomass (OD_600_ = 1.7, Fig. 2). This result reveals its great tolerance ability, fast degradation speed, and high utilization efficiency for *n-*alkanes.

### Sequence analysis and alignment of the alkane-degradation genes in strain SJTD-1

**Figure 5:**
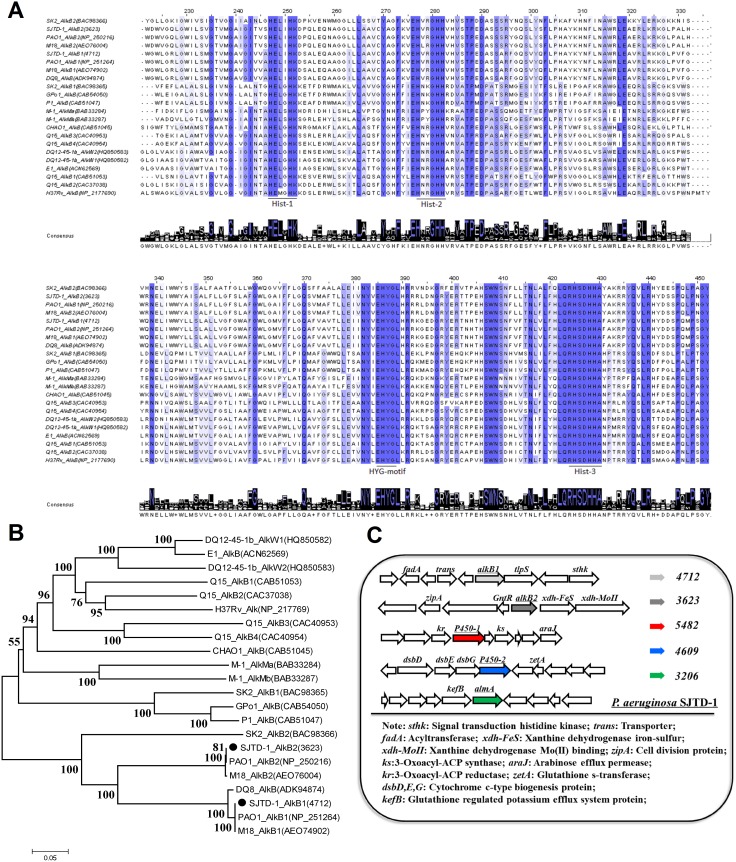
Sequence alignment of alkane hydroxylases AlkB1 and AlkB2 from several strains and genetic distribution of five putative alkane hydroxylases in *P. aeruginosa* SJTD-1. Strains list: SJTD-1, *P. aeruginosa* SJTD-1; PAO1, *P. aeruginosa* PAO1; DQ8: *P. aeruginosa* DQ8; GPo1, *P. putida* Gpo1; P1, *P. putida* P1; CHAO: *P. fluorescens* CHAO; SK2, *Alcanivorax borkumensis* SK2; M-1, *Acinetobacter sp.* M-1; Q15, *Rohodococcus erythropolis* Q15; H37Rv, *Mycobacterium tuberculosis* H37Rv; DQ12-45-1b, *Dietzia sp.* DQ12-45-1b; E1, *Dietzia sp.* E1. (A) The conserved amino acid residues in all the sequences are indicated with a blue background, and the dark degree is in direct proportion to the degree of conservation. The three conserved histidine boxes (Hist-1, Hist-2, and Hist-3), and one HYG motif are underlined. The conserved amino acid associated with each position is shown below the alignment (created by Clustal Origin). (B) Phylogenetic relationship based on the complete amino acid sequences of AlkB1 and AlkB2 from *P. aeruginosa* SJTD-1 and other published AlkB amino acid sequences. The phylogenetic tree was determined using the Kimura two-parameter distance model, and bootstrap analysis was performed with 1,000 repetitions using MEGA 5.0. (C) Schematic profile of the genomic organization of AHs homologues in *P. aeruginosa* SJTD-1. *alkB1* and *alkB2*, alkane-1 monooxygenases (lawngreen and grey, separately); *P450-1* and *P450-2*, cytochrome P450s (red and blue, respectively); *almA*, AlmA-like monooxygenase (green); *tlpS*, methyl-accepting chemotaxis protein.

The whole-genome DNA sequence of strain SJTD-1 has been obtained and deposited in GenBank under the accession number AKCM00000000 [23]. It was also annotated by the RAST server. Two putative genes encoding AlkB monooxygenases were found to be involved in the first step of alkane degradation. Gene 4712 (*alkB*1) encodes a putative AlkB1 monooxygenase, comprising 383 amino acids, and with an estimated molecular weight of 42.1 kDa. The second *alkB* gene 3623 (*alkB*2) encodes AlkB2 monooxygenase, comprising 378 amino acids and with an estimated mass of 41.6 kDa. The sequence of *alkB*1 and *alkB*2 genes exhibited 78% identity, and their derived proteins exhibited 68% identity. The cross-genera sequence alignments of AlkB1 and AlkB2 with other published AlkB sequences indicated that all of these alkane hydroxylases share one conserved HYG motif and three highly conserved regions of the Hist boxes containing eight histidines [30]–[32] (Fig. 5A).

To further understand the relationship between AlkB proteins, phylogenetic analysis was performed based on the characterized AlkB monooxygenases from *Pseudomonas*, *Alcanivorax*, *Acinetobacter*, *Mycobacteria,* and *Rhodococci* (Fig. 5B). In the phylogenetic tree, the two AlkB proteins from *P. aeruginosa* PAO1 and M18 were clustered as alkane monooxygenase 1, and these were close to the alkane monooxygenases from other gram-negative bacteria, such as *Alcanivorax borkumensis* SK2 and *Acinetobacter sp* M-1. In fact, the AlkBs of strain SJTD-1 showed very high sequence identity with other *Pseudomonas* AlkBs. For instance, the identity of SJTD-1 AlkB1 to *P. aeruginosa* PAO1 and M18 AlkB1 was 100%, and the AlkB2 identity was 100% and 99%, respectively. Another reported *Pseudomonas* strain DQ8, that could easily break down alkanes and polycyclic aromatic hydrocarbons harbored only one AlkB homologue, which was mapped to SJTD-1 AlkB2 with 100% identity [8]. In addition, two cytochrome P450 monooxygenase homologues (gene 5482 and 4609) and one AlmA-like monooxygenase homologue (gene 3206) were also found in the genome of strain SJTD-1. Sequence analysis showed that genes *P450-1* (gene 5482) and *P450-2* (gene 4609) encoded a 418-amino-acid-protein and a 444-amino-acid-protein, respectively; they shared 99% amino acid sequence identity to those of *P. aeruginosa* PAO1 and 26% to those of *Alcanivorax dieselolei* B-5 [33]. As for the putative *almA*-like gene, its protein (499 aa) displayed 56% identity with the putative flavin-containing monooxygenase of *A. dieselolei* B-5, and only 50% identity to the AlmA of *Acinetobacter sp.* DSM 17874 [34].

As shown in Fig. 5C, the genomic organization of the five alkane hydoxylases, none of which are located within the gene cluster encoding the genes responsible for alkane catabolism like those seen in *P*. *putida* GPo1 and other hydrocarbonoclastic bacteria [19], [33], [35]. It should be noted that the surrounding open reading frames of the *alkB* genes in strain SJTD-1 were different from those in other strains, while the rubredoxin (Rd)-encoding genes, the rubredoxin reductase-encoding genes, and the transcriptional regulatory protein-encoding genes were often located immediately downstream of the *alkB* gene. No rubredoxin reductase-encoding gene was sought out; and no fusion Rd domain was found near the *alkB* genes, as opposed to that observed in *Dietzia sp.* DQ12-45-1b [31]. However, some important clues were found despite the decentralized distributions over the chromosome [36]. One exception was *tlpS*, a gene coding for a methyl-accepting chemotaxis protein that is potentially involved in chemotaxis towards long-chain *n-*alkanes. It was found located downstream of *alkB*2 [37]. A putative transcriptional regulator of the GntR family was located upstream of AlkB2 and the intergenic region between *alkB2* and *gntR* was only 181 bp. Therefore, it is conceivable that in strain SJTD-1, *n-*alkanes may be utilized through a more diversified pattern than that in other reported hydrocarbonoclastic bacteria.

### Phenotypic study of the putative alkane hydroxylation genes in strain SJTD-1

**Figure 6:**
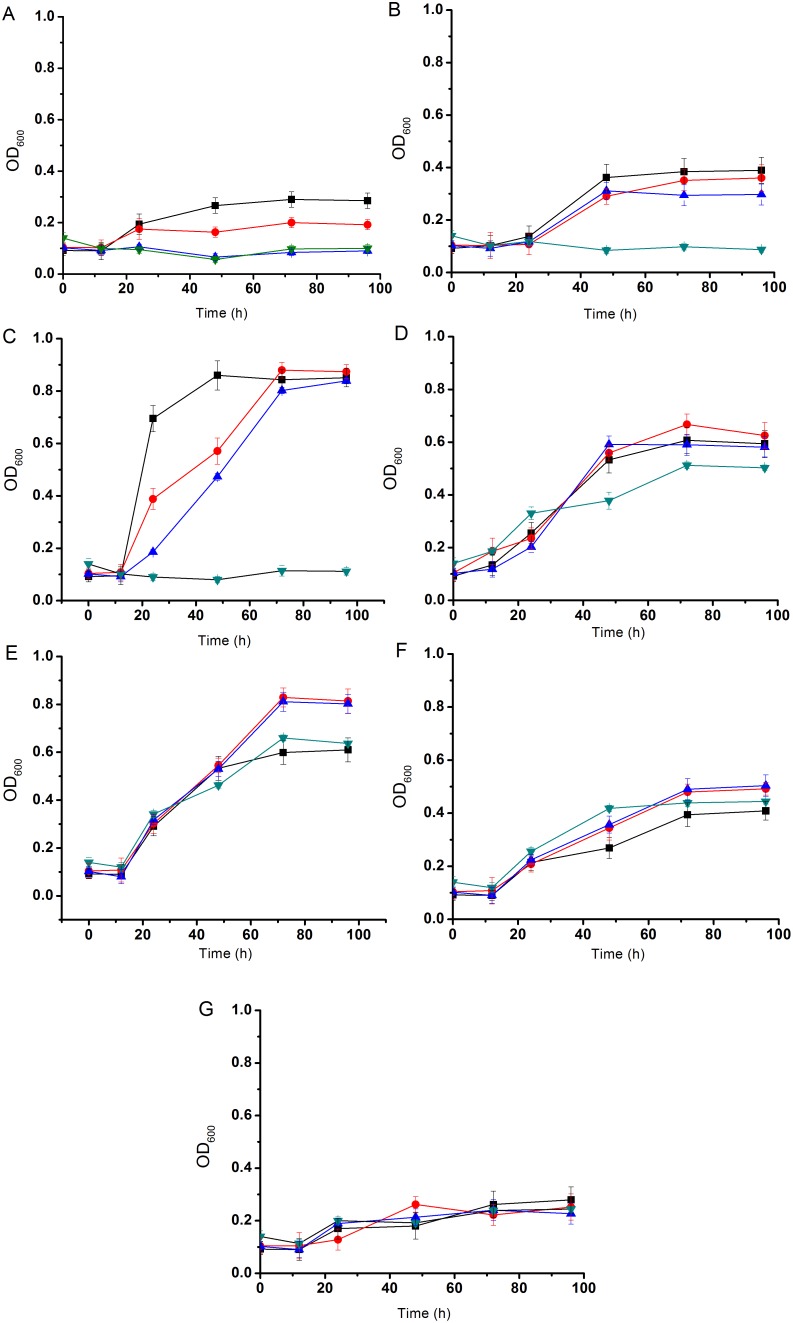
Growth curves of *P. aeruginasa* SJTD-1 (▪), Mut*_alkB1_* (•), Mut*_alkB2_* (▴), and Mut*_alkB1&2_* (▾) in *n-*alkanes. The strains were inoculated in BSM supplemented with 500 mg/L *n-*alkanes: *n*-dodecane (A), *n*-tetradecane (B), *n*-hexadecane (C), *n*-octadecane (D), *n*-eicosane (E), *n*-docosane (F), and *n*-tetracosane (G) and cultured for seven days at 30°C.

**Figure 7:**
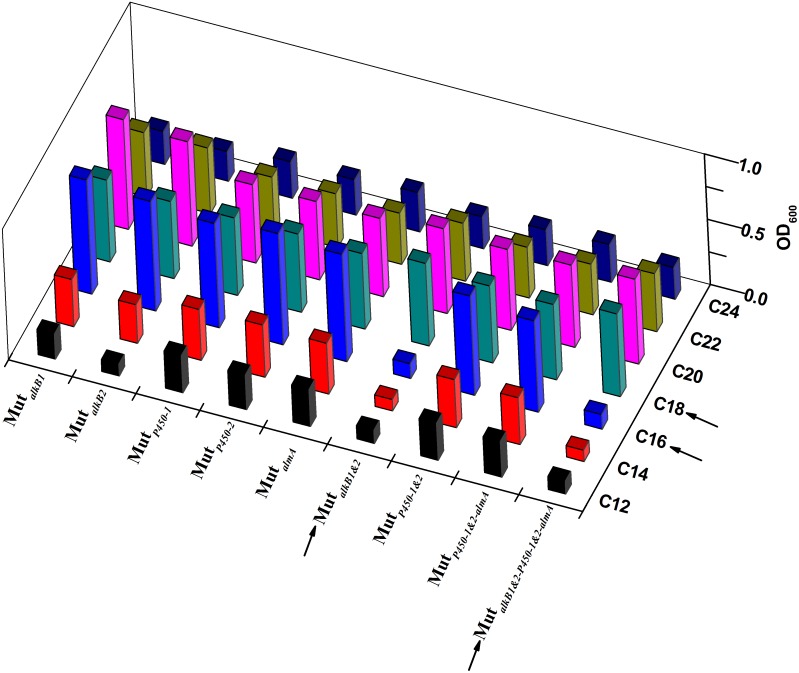
3D-bar growth representations of all the defective mutants with C_12_–C_24_ *n-*alkanes. Values on the horizontal axis profile the genotype of the mutants with single or multiple deletions of alkane hydroxylases; The Y-axis specifies the tested *n-*alkanes, coloring from black to dark blue black. The Z-axis of bar altitudes represents the OD_600_ for the viability of each mutant. Sharp contrasts were symbolized using arrows when both *alkB1* and *alkB2* were knocked out, and cultivated in C_16_ and C_18_ alkane media, respectively.

In order to determine the functions of the two *alkB* genes in the *n*-alkane oxidative process and in order to understand their preferences to *n-*alkanes, we knocked out the two genes separately and simultaneously, and then monitored the utilization capability of the three mutants (Mut*_alkB1_*, Mut*_alkB2_*, and Mut*_alkB1&2_*) for the C_12_–C_24_ *n-*alkanes. The mutants were verified using PCR detection (Fig. S1). For the medium-chain *n-*alkanes (C_12_–C_16_), single-knockout mutants showed an obvious delay as compared to the wild type SJTD-1, and double-knockout mutants could not utilize the *n-*alkanes at all (Fig. 6A–6C). It meant that these two genes were responsible for the degradation of C_12_–C_16_ *n-*alkanes and that both of them were involved in the oxidative process. The *alkB2* defect showed a more pronounced effect on the cell growth than did the *alkB1* defect, thereby suggesting that AlkB2 played a more major role in the oxidation of medium-chain alkanes (Fig. 6A–6C and Fig. 7). Unexpectedly, the poor viability of Mut*_alkB1&2_* recovered to a normal state when C_18_–C_24_ alkanes were used as the sole source of carbon. No obvious growth decrease was found in the single-knockout mutants (Fig. 6D–6G). This implied that there were some hitherto unknown genes involved in the utilization of longer-chain *n-*alkanes.

We further studied three more potential alkane hydroxylases (AH) genes (two putative cytochrome *P450* genes and one *almA* monooxygenase gene) listed in Table 1. In order to judge the physiological role of each putative AH, single-knockout, double-knockout or multi-knockout mutants were obtained, and their viabilities were evaluated in the BSM media supplemented with *n-*alkanes (Fig. 7). On the whole, the divergence of the mutants’ viabilities in Fig. 7 resulted from the dissimilar enzymatic preferences of AHs within the range of the substrate spectrum. Strains with absence of two AlkBs almost failed to grow on C_12_–C_16_ alkanes; whereas P450 and AlmA made a minor contribution to the degradation of these substrates, as their defective strains still grew to a comparable level relative to the parent. However, it could be concluded that the recovery of viability originated from the transcription of P450 and AlmA oxygenases, as mutant Mut*_alkB1&2-P450-1&2-almA_* showed a similar growth level as that of the wild type for C_18_–C_24_. Therefore, an affirmative hypothesis for the contribution of other undefined AHs to microbial catabolism of long-chain alkanes (C_18_–C_24_) was made. So far, these findings explain the metabolic roles of identified AHs within their preferred substrate range and further provided clues for exploring the uncharacterized alkane hydroxylases.

### Transcriptional expression profiles of the *alkB1*, *alkB2*, *p450* and *almA* genes in strain SJTD-1

**Figure 8:**
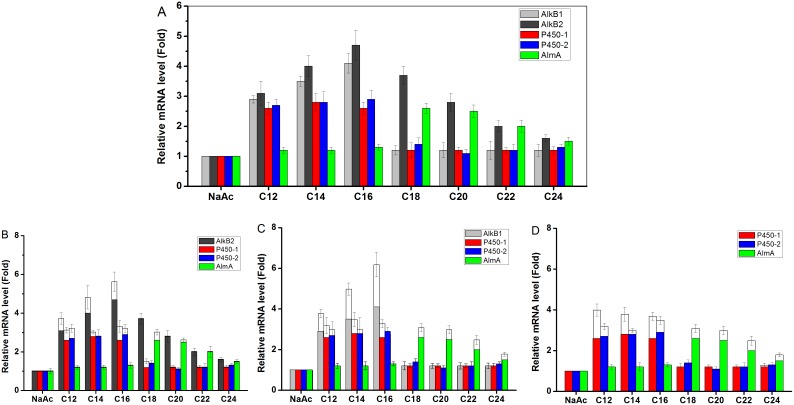
Transcriptional levels of five putative AHs genes in wild type SJTD-1 and in three knockouts. Relative expression levels were determined with real-time RT-PCR. *16S rRNA* gene was used as the reference gene. The expression levels of genes in strain SJTD-1 (A), strain Mut*_alkB1_* (B), strain Mut*_alkB2_* (C), and strain Mut*_alkB1&2_* (D) were shown. All strains were grown in different *n-*alkanes (C_12_ to C_24_ alkanes as the sole carbon source), and cells grown with sodium acetate were used as controls. Standard errors were calculated from three independent determinations.

Five putative AHs in strain SJTD-1 were revealed as expected, and the diversity of AHs allowed for a rapid adaptation of SJTD-1 to the changing environments of various alkanes. To articulate the contribution of each AH to the utilization of alkanes, their transcriptional profiles were investigated. Fig. 8A shows that C_12_–C_24_ alkanes rendering the viability of SJTD-1 rested with a universal expression of AHs in any case. AlkB1 showed a relatively narrow inducer range of C_12_–C_16_ than did AlkB2, which could be induced by C_12_–C_24_ alkanes, thereby eliciting more than a two-fold change on its transcriptional level (Fig. 8A). Two P450 monooxygenases probably contributed to the mineralization of C_12_–C_16_ alkanes, because of the 2.5-fold change with those substrates. However, when C_18_–C_24_ alkanes were used, their transcriptional changes fell down sharply (Fig. 8A). The AlmA-like oxidase was expressed at a low level (1.5–3 fold change), only when the C_18_–C_24_ *n-*alkanes were used (Fig. 8A). So far, it was facile to explain that the abundant transcription of AHs in the hexadecane-induced cells lead to the most significant utilization of *n*-hexadecane during SJTD-1 growth in Fig. 2.

As the single deletion of the five AHs made little impact on the cell growth, the transcription levels of the targeted AHs were analyzed in the *alkB1*-defective, *alkB2*-defective, and double-deletion mutants. A functional complementation of AHs was observed during the cultivation period. As illustrated in Fig. 8B and 8C, the transcriptional level of an alternative AlkB hydroxylase was enhanced and it was thus responsible to terminally oxidize *n-*alkanes, particularly when one AlkB was absent. This terminal oxidation of *n-*alkanes was limited as indicated by the overlapping area of both AlkB substrate spectrums, i.e. C_12_–C_16_ alkanes. Among these substrates, two P450s also displayed changes in expression and contributed to the complementary effects of AlkBs. Interestingly, the AlmA-like oxygenase increased its transcription to some extent (within the range of C_18_–C_24_ alkanes) when *alkB2* was removed rather than *alkB1* (Fig. 8B–8D). Therefore, the less attenuated viability of strain SJTD-1 on alkanes even when any alkB gene was inactivated can be attributed to the upregulation of functionally-related AHs and their metabolic contribution.

As mutants lacking both the AlkB hydroxylases showed poor propagation on C_12_–C_16_ alkanes, but did not affect the duplication rate on the C_18_ alkane, the transcription of two P450 and AlmA-like oxygenases was surveyed as depicted in Fig. 8D. However, no obvious alteration was found for these AHs in the single or double *alkB* mutants. Mutants with a consecutive deletion of five AHs still survived on *n*-octadecane and also duplicated to a normal level of OD_600_ (Fig. 7). This strongly suggested that the biodegradation of C_18_–C_24_ alkanes differed from that of the C_12_–C_16_ medium chain alkanes, and that a cluster of unidentified AHs may participate in this process, by transforming the long chain alkanes into lower hydrocarbons.

## Discussion

*P. aeruginosa* strain SJTD-1, a new soil isolate, was determined as a hydrocarbonoclastic consumer of medium- and long-chain alkanes (C_12_–C_30_). Based on the 16S rDNA sequence alignments and phenotype analysis, strain SJTD-1 showed close homogeneity to the well-documented *P. aeruginosa* PAO1 and *P. aeruginosa* DQ8. However, strain SJTD-1 exhibited no human pathogenicity like PAO1, and it degraded polycyclic aromatic hydrocarbons only to some extent, unlike DQ8. In fact, a relatively narrower substrate range of C_12_–C_24_ supported a sustainable growth of strain SJTD-1. For this range of *n-*alkanes, strain SJTD-1 showed outstanding degradation efficiency and utilization speed. *n*-Hexadecane followed by *n*-tetradecane and *n*-octadecane were assimilated as preferential carbon sources for this strain as these hydrocarbons could be rapidly biodegraded by SJTD-1. *n-*Tetradecane, *n-*hexadecane, and *n-*octadecane (at a concentration of 500 mg/L) were completely degraded within 36 h. SJTD-1 could also tolerate and degrade 2.0 g/L *n-*hexadecane within three days. As compared to other alkane-utilizing strains, strain SJTD-1 degrades the same type and same amount of *n-*alkanes more efficiently and more quickly. Most of the alkane-consuming strains mineralize equivalent amounts of alkanes at a much slower rate and require a much longer degradation time (Table 2). When 500 mg/L of *n*-octadecane was depleted by strain SJTD-1 in 36 h, *Alcanivorax sp.* 2B5 simply consumed 16.07% of this substrate at the beginning of 48 h. In contrast to *P. aeruginosa* DQ8, strain SJTD-1 showed a comparable transformation velocity but the initial concentration of *n*-tetradecane was 5-fold higher in this case.

**Table 2:** Comparison of alkane degradation by different strains.

Strain	Substrates	Conservation rate	Sources/Reference
*P. aeruginosa* SJTD-1	C_14_, C_16_, C_18_	>99% in 36 h	This study
	500 mg/mL		
*P. aeruginosa* DQ8	C_14_, 100 mg/L	>99% in 24 h*	[8]
*Alcanivorax sp.* 2B5	C_18_, 500 mg/L	16.07% in 48 h	[32]
		>99% in 96 h	
*Acinetobacter*	C_16_, 400 mg/L	>99% in 60 h	[39]
*Dietzia* DQ12-45-1b	C_16_, 2.5 mg/mL	31% in 21 d	[11]

*with 0.005% yeast extract in the medium.

In order to decipher the mechanism by which SJTD-1 breaks down the *n-*alkanes, the initial but rate-limiting step deserves special attention. This step marks the beginning of the terminal oxidation catalyzed by alkane hydroxylases. The genome sequencing results indicated that at least five alkane hydroxylases in strain SJTD-1 were involved in the oxidization process. Through transcriptional analysis, two *alkB* genes were found to be greatly induced by *n-*alkanes, with AlkB2 sharing a broader substrate range. The two P450 monooxygenases were induced only when medium-length alkanes were used, whereas the C_18_–C_24_ alkanes were able to regulate the expression of the AlmA oxygenase, which is known to be involved in the oxidation of super long-chain alkanes (>C_30_) [34]. The fact that *n*-hexadecane showed a prominent growing effect for strain SJTD-1 was evident from the topmost transcription of AHs during hexadecane-induced culture, as compared to the other *n-*alkanes. A transcriptional enhancement of two AlkBs was evident when either of the AlkBs was inactivated, strongly suggesting their overlapping substrate spectrum and functional identities.

In order to specify the roles of AHs in the alkane degradation, some important defective strains were deliberately constructed and their phenotypic profiles were represented under the given range of C_12_–C_24_ alkanes. For medium chain alkanes (C_12_–C_16_), AlkB hydroxylases showed a dramatic performance, and bacterial growth was seriously influenced when the two alkB genes were deleted. Moreover, the defective strain in absence of AlkB2 grew at a slower rate than the AlkB1 defective mutant did. It was therefore speculated that AlkB2 played a central role in the catabolism of C_12_–C_16_ alkanes, which was more important than the role played by AlkB1. The other three AHs including P450 and AlmA oxygenases had little effect on *n*-alkane degradation, although their transcription would somewhat offset the deletion of the *alkB* genes. This implies a heavy redundancy of AHs for the microbial degradation of *n-*alkanes. However when C_18_–C_24_ *n*- alkanes were used, strain SJTD-1 utilized another set of alkane hydroxylases to metabolize these *n-*alkanes. Therefore, it is important to understand how strain SJTD-1, in the absence of all known AHs, still manages to survive on the long-chain alkanes.

The key factors involved in the degradation of alkanes, such as AlkB, rubredoxin, and other related alkane hydroxylases, showed a scattered distribution in the genome of strain SJTD-1, without the occurrence of gene clusters as represented in *Pseudomonas putida* cells. This was also observed among other *P. aeruginosa*, such as *P. aeruginosa* PAO1 and *P. aeruginosa* RR1. However, their sporadic distribution did not compromise the bacterial utilization of *n-*alkanes. This indicates that a compensatory mechanism was responsible for the regulation and coordination of these multi-step catalytic reactions. For instance, it has been proposed that genes encoding two significant electron transfer proteins, viz. rubredoxin and rubredoxin reductase, were distant from the inducible alkB genes and constitutively expressed in any case [38]. To better understand the mechanism behind this disordered genetic organization, we need to resort to a comprehensive approach, such as an iTRAQ-based proteomics study, which would systematically characterize the global response of strain SJTD-1 to various types of medium- and long-chain *n-*alkanes.

## Supporting Information

Figure S1 **PCR verification of the mutant strains.** The genomic DNA of all the mutants as well as the wild type *P. aeruginosa* SJTD-1 was extracted and amplified with the test primers listed in Table S1. The colonies with shorter PCR products (400–500 bp) represented successful deletions. (A) The PCR products of Mut*_alkB1_*, Mut*_alkB1&2_*, and wild type SJTD-1; (B) The PCR products of Mut*_almA_* and wild type SJTD-1; (C)The PCR products of Mut*_P450-1_*, Mut*_P450-2_*, Mut*_P450-1&2_*, and wild type SJTD-1.
(TIF)

Table S1 **List of primers used in this work.** All primers are listed from 5′ to 3′. The F and R primers represent forward and reverse primers, respectively.
(DOCX)

Text S1 **Nucleotide and amino acid sequences of five Alkane Hydroxylases (AHs) in** ***P. aeruginosa*** **SJTD-1.** The five AHs are AlkB1, AlkB2, P450-1, P450-2 and AlmA-like. Gene numbers presented here are the numbers annotated in RAST server.
(DOCX)
