# Supplementary figures and images for: Characterization of the Medium- and Long-Chain n-Alkanes Degrading Pseudomonas aeruginosa Strain SJTD-1 and Its Alkane Hydroxylase Genes
Source: PLoS One. 2014 Aug 28;9(8):e105506. doi: 10.1371/journal.pone.0105506 (PMC4148322; doi:10.1371/journal.pone.0105506)

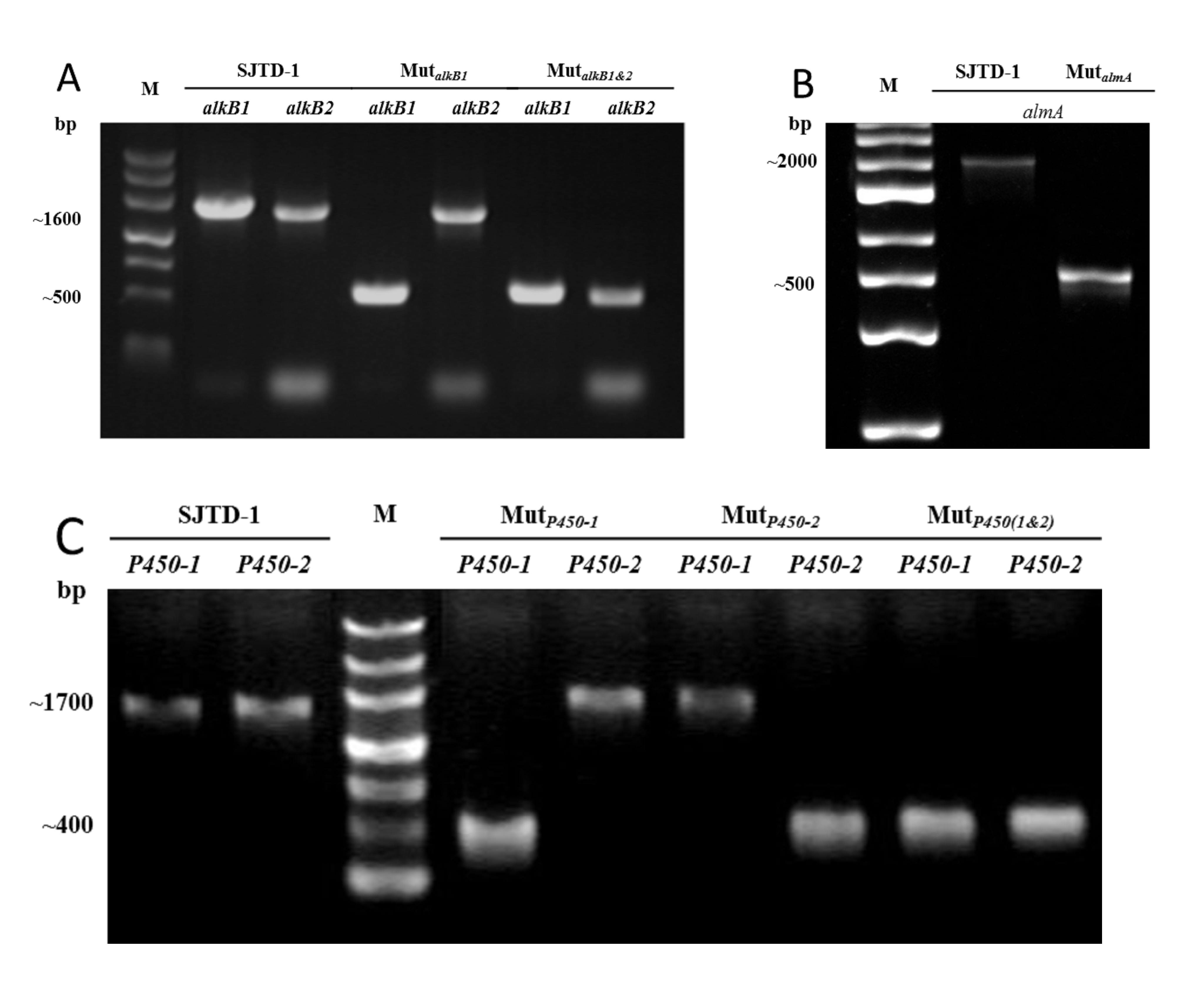

Supplement: Figure S1 — PCR verification of the mutant strains. The genomic DNA of all the mutants as well as the wild type P. aeruginosa SJTD-1 was extracted and amplified with the test primers listed in Table S1. The colonies with shorter PCR products (400–500 bp) represented successful deletions. (A) The PCR products of MutalkB1, MutalkB1&2, and wild type SJTD-1; (B) The PCR products of MutalmA and wild type SJTD-1; (C)The PCR products of MutP450-1, MutP450-2, MutP450-1&2, and wild type SJTD-1. (TIF) [file pone.0105506.s001.tif]
