# Supplementary material for: Characterization of the Medium- and Long-Chain n-Alkanes Degrading Pseudomonas aeruginosa Strain SJTD-1 and Its Alkane Hydroxylase Genes
Source: PLoS One. 2014 Aug 28;9(8):e105506. doi: 10.1371/journal.pone.0105506 (PMC4148322; doi:10.1371/journal.pone.0105506)
Supplement: Table S1 — List of primers used in this work. All primers are listed from 5′ to 3′. The F and R primers represent forward and reverse primers, respectively. (DOCX) [file pone.0105506.s002.docx]

**Table S1 List of primers used in this work**

| Name | Sequence^*^ |
| --- | --- |
| 16S-seq-F | gagcggataacaatttcacacagg |
| 16S-seq- R | cgccagggttttcccagtcacgac |
| 3623-HR-F1 | aatctaacaagataaatacgaggacttcccatgaacatcaaaaagtttgcaaaac |
| 3623-HR-R1 | cgaaggtgccgtttcccaggggcgggacgcttaggtggcggtacttgggt |
| 3623-HR-F2 | aggccggccaagggaggacaattgtcagacaatctaacaagataaatacgaggac |
| 3623-HR-R2 | gtcagtttgcccataaacgaaaacggcacccgaaggtgccgtttccca |
| 4712-HR-F1 | aggattgtcggacaaaaactggagcgcacgatgaacatcaaaaagtttgcaaaac |
| 4712-HR-R1 | tcgggcacggagcggggacgccggccattcttaggtggcggtacttgggt |
| 4712-HR-F2 | ggcagcccggctcggtgatggttcgattgtaggattgtcggacaaaaactggagc |
| 4712-HR-R2 | caaggcgggattgcccgaacgaagagctattcgggcacggagcgggga |
| 5482-HR-F1 | ccgccgccccggcggcgaaggaggagcaacatgaacatcaaaaagtttgcaaaac |
| 5482-HR-R1 | gcaggctgtggacaagaatttccttggcatttaggtggcggtacttgggt |
| 5482-HR-F2 | tgatcgacgggatgttccgccccatcgtcaccgccgccccggcggcgaag |
| 5482-HR-R2 | agccacgggcatcgccgttttccaggaggcgcaggctgtggacaagaatt |
| 4609-HR-F1 | gcgttacgccaacccagcggagcaagggccatgaacatcaaaaagtttgcaaaac |
| 4609-HR-R1 | ccgcagcgagggcagccggctgatcctgcgttaggtggcggtacttgggt |
| 4609-HR-F2 | gcgccgagccgccggccatctatactccctgcgttacgccaacccagc |
| 4609-HR-R2 | caacgcctatcacgaatacggcgcacgcaaccgcagcgagggcagccg |
| 3206-HR-F1 | gctggacgcttccgccgatcgaggaaccttatgaacatcaaaaagtttgcaaaac |
| 3206-HR-R1 | tggcgaacgcctgagcgacgcccaccgggtttaggtggcggtacttgggt |
| 3206-HR-F2 | gtagggtggagggcatcagcccagcagacggctggacgcttccgccgat |
| 3206-HR-R2 | acgcctggcgcaaggcctacgggcaggtcgtggcgaacgcctgagcga |
| 3623-T-F | tccggggccttgaacgtcat |
| 3623-T-R | cgcaggtagtcgaggacg |
| 4712-T-F | ggcagcgagagacggcca |
| 4712-T-R | atcatgtcgccttccatgtg |
| 5482-T-F | cgcaacagcgcgaccaag |
| 5482-T-R | acgtcggtgaagcgcacg |
| 4609-T-F | ctgatggcgtcgatgggc |
| 4609-T-R | ttggcggctcgggttgtc |
| 3206-T-F | ggagaccccggagaaacctt |
| 3206-T-R | gaacaggggcaattgccgg |
| 3623-RT-F | ggatctggctgatcgcggtgctc |
| 3623-RT-R | cgcggtagtagccctgtgcttcc |
| 4712-RT-F | ggctctggctgctgctggcg |
| 4712-RT-R | cgtaatagccctggccgagcaact |
| 5482-RT-F | gatctcgccactgcacgcgc |
| 5482-RT-R | gtggctgagtccctcgccgat |
| 4609-RT-F | tgatccgctcccgtgcgccg |
| 4609-RT-R | cgtagaagcgcgccagcgg |
| 3206-RT-F | acgggagaacggcatcgaccg |
| 3206-RT-R | ggcgaagtcctcgcgaccgac |
| 16S-RT-F | gcctaccaaggcgacgatccgt |
| 16S-RT-R | ccatgccgcgtgtgtgaagaag |

^*^ All primers are listed from 5’ to 3’. The F and R primers represent forward and reverse primers, respectively.
