# Supplementary material for: Characterization of the Medium- and Long-Chain n-Alkanes Degrading Pseudomonas aeruginosa Strain SJTD-1 and Its Alkane Hydroxylase Genes
Source: PLoS One. 2014 Aug 28;9(8):e105506. doi: 10.1371/journal.pone.0105506 (PMC4148322; doi:10.1371/journal.pone.0105506)
Supplement: Text S1 — Nucleotide and amino acid sequences of five Alkane Hydroxylases (AHs) in P. aeruginosa SJTD-1. The five AHs are AlkB1, AlkB2, P450-1, P450-2 and AlmA-like. Gene numbers presented here are the numbers annotated in RAST server. (DOCX) [file pone.0105506.s003.docx]

**Characterization of *Pseudomonas aeruginosa* strain SJTD-1 with the capability of degrading medium- and long-chain *n*-alkanes, and analysis of its alkane hydroxylase genes**

Huan Liu, Jing Xu, Rubing Liang*, Jianhua Liu*

State Key Laboratory of Microbial Metabolism, School of Life Sciences and Biotechnology, Shanghai Jiaotong University, Shanghai 200240, China

***Corresponding author:**

Rubing Liang, Ph.D.

E-mail: [icelike@sjtu.edu.cn](mailto:icelike@sjtu.edu.cn)

Jianhua Liu, Ph.D.

E-mail: [jianhualiudl@sjtu.edu.cn](mailto:jianhualiudl@sjtu.edu.cn)

**Text S1 Nucleotide and amino acid sequences of five Alkane Hydroxylases (AHs) in *Pseudomonas aeruginosa* SJTD-1**

Gene 4712 [Alkane-1 monooxygenase (EC 1.14.15.3)]

atgtttgaaaatttctctcccagcaccatgctggccatcaagaagtacgcctattggctctggctgctgctggcgttgtccatgccgttcaactactggatggcccaggacagcgcacatccggccttctgggcattttccctggtgattgcggtattcggcatcgggccgctgctggacatgctgttcggccgcgacccggccaaccccgacgaggaaacccagacgccgcagttgctcggccagggctattacgtattgctgaccctggccacggtgccggtgctcatcggtacgctggtgtgggccgctggcgtgttcgtcgctttccaggagtggggctggctcggccggttgggctggatcctctcgatgggcacggtgatgggagcggtggggatcgtcgtcgcccatgagctgatccacaaggactcggcgctggagcaggctgccggcggcatcctgctggccgccgtgtgctacgcggggttcaaggtcgagcatgtgcgcggccaccatgtgcatgtgtctacgccggaggacgcttcgtcggcgcgtttcggccagtcggtctaccagttcctgccgcatgcctacaagtacaacttcctcaacgcctggcgccttgaagcggtgcggctgcgcaagaagggcctgccggtgttcggctggcagaacgaactgatctggtggtacctgctgagcctggcgttgctggtcggtttcggttgggcgttcggctggctggggatggttttcttccttggccaagcgttcgtcgcggtgacactgctggagatcatcaactacgtcgagcactacggcctgcatcggcgaaagggcgaggacgggcgctacgagcggaccaaccatacccactcctggaacagcaacttcgtcttcaccaacctggtcctgttccatctgcaacggcattccgaccatcatgccttcgccaaacgcccctatcaggtcctccgtcattatgacgatagcccgcagatgcccagcggttatgccgggatggtggtgctggcgctgatcccgccgctgtggcgggcggtcatggaccccaaggtgcgggcctactatgcgggcgaagagttccagttgacggccgaacagagcgagcggccggcagcttcctga

MFENFSPSTMLAIKKYAYWLWLLLALSMPFNYWMAQDSAHPAFWAFSLVIAVFGIGPLLDMLFGRDPANPDEETQTPQLLGQGYYVLLTLATVPVLIGTLVWAAGVFVAFQEWGWLGRLGWILSMGTVMGAVGIVVAHELIHKDSALEQAAGGILLAAVCYAGFKVEHVRGHHVHVSTPEDASSARFGQSVYQFLPHAYKYNFLNAWRLEAVRLRKKGLPVFGWQNELIWWYLLSLALLVGFGWAFGWLGMVFFLGQAFVAVTLLEIINYVEHYGLHRRKGEDGRYERTNHTHSWNSNFVFTNLVLFHLQRHSDHHAFAKRPYQVLRHYDDSPQMPSGYAGMVVLALIPPLWRAVMDPKVRAYYAGEEFQLTAEQSERPAAS

Gene 3623 [Alkane-1 monooxygenase (EC 1.14.15.3)]

Atgtttgcctcgctttcctctgcctggatgctgcgtctgaaaaagtacggctactggatctggctgatcgcggtgctcggcatcccgctcagctactggtggtcgctcggtagcgactaccccaacgcctggccctggctggtgatcagcgtggtgttcgggctgatcccgatcctcgatgccatcgtcggccgcgatccggccaaccccgaggaagccagcgaagtgccggagatggaagcacagggctactaccgcgtactgtccctggccaccgtcccgctgttgctgggcatgctcgtctggtccggctggatcctcgcccacgagacccgctgggactgggtcggccaactgggctggatcctgtcggtgggcaccgtgatgggcgccatcggcatcaccgtctcccacgaactgatccacaaggacccgcaactggaacagaacgccggcggcctgctgctggcagcggtgtgctatgccggcttcaaggtcgaacacgtgcgcggccaccatgtacacgtctcgaccccggaagatgcctcgtcctcgcgctacggccagagcctctactcgttcctcccgcacgcctacaagcacaacttcctcaacgcctggcgcctggaggccgagcgcctgaagcgcaagggcctgccggccctgcactggcgcaacgagctgatctggtggtacgccatcagcgccctcttcctgctcggcttcagcctggccttcggctggctgggagcgatcttcttcctcggccagtcggtgatggccttcaccctgctggagatcgtcaactacgtcgagcactacggcctgcatcggcggcgcctggacaacggccgctacgaacgcaccacgccggaacactcgtggaacagcaatttcctcctgaccaacctgttccttttccacctgcagcgccattccgaccaccatgcctacgccaagcgccgctaccaggtgctgcgccactacgacagcagcccgcaactgcccaacggctacgccgggatgatcgtcctcgccctgttcccgccgctctggcgcgcggtgatggacccgaaggtgcgcgcctactatgccggcgaggaataccagcttaccgacacccagcgcatctga

MFASLSSAWMLRLKKYGYWIWLIAVLGIPLSYWWSLGSDYPNAWPWLVISVVFGLIPILDAIVGRDPANPEEASEVPEMEAQGYYRVLSLATVPLLLGMLVWSGWILAHETRWDWVGQLGWILSVGTVMGAIGITVSHELIHKDPQLEQNAGGLLLAAVCYAGFKVEHVRGHHVHVSTPEDASSSRYGQSLYSFLPHAYKHNFLNAWRLEAERLKRKGLPALHWRNELIWWYAISALFLLGFSLAFGWLGAIFFLGQSVMAFTLLEIVNYVEHYGLHRRRLDNGRYERTTPEHSWNSNFLLTNLFLFHLQRHSDHHAYAKRRYQVLRHYDSSPQLPNGYAGMIVLALFPPLWRAVMDPKVRAYYAGEEYQLTDTQRI

Gene 5482 [putative cytochrome P450 hydroxylase]

gtgcctgatcgcaaactgagactgggcgaggaactgatctcgccactgcacgcgctctacgacggcctgcaggtggacggcgcgccgcgtcccgcgcatcgcgccgccgagcatccggtgtgggtggtgacgcgctaccgcgacgcgcgcaaggtcctcaaccatccgggcgtccgccgcgacgcccggcaggccgccgaactctacgcgaagcgtaccggcagcccgcgcgcggggatcggcgagggactcagccaccacatgctcaacctcgacccgccggaccatacccgcctgcgctcgctggttggccgcgcgttcaccccgcgccaggtggagcgcctgcaaccgcatatagaacggatcaccgaggcattgctggacgccatggccggccgcgaacaggccgacctgatggccgacttcgcgatcccgctgaccatcgcggtgatcttcgagctgctgggcattcccgaggccgagcgcgaacacgcccgccagtcctgggagcgccaggcggaactgctgtcgccggaggaggcccaggccctggccgatgcgcaggtcgactacctgcgcgtgctgctcgaggccaagcgccggcagccggccgacgacgtctacagcgggctggtgcaggccgccgacgagagcggccagttgagcgaagcggaactcgtctccatggcccacctgctgatgatgagcggcttcgagaccaccatgaacatgatcggcaacgcgctggtcaccctgctggtcaacccgcagcaactaacgttgctgcgggcgcagccggaactcctgcccaacgccatggaagaactggtccgccacgacagtccggtgcgcgcctcgatgttgcgcttcaccgtggaagacgtggaactggacggggtcaccattcccgccggcgaatacatcctggtctccaacctgaccgccaaccacgatgccgagcgcttcgacgatcccgaccgcctcgacctcacccgcaacaccgatggccatctcggctacggcttcggcgtgcactactgcgtcggcgcctcgctggcccggctggaggggcggatcgccatccagcgcctgctcgcgcgcttccccgacctccagttggcggtgccccacgcggagctgcagtggctgccgatcaccttcctccgcgccctgatcagcgtgccggtgcgcaccggatgcagcgccccggcgaacaccgcctcccacgccaacccgatcgagaggatcgcccaatga

MPDRKLRLGEELISPLHALYDGLQVDGAPRPAHRAAEHPVWVVTRYRDARKVLNHPGVRRDARQAAELYAKRTGSPRAGIGEGLSHHMLNLDPPDHTRLRSLVGRAFTPRQVERLQPHIERITEALLDAMAGREQADLMADFAIPLTIAVIFELLGIPEAEREHARQSWERQAELLSPEEAQALADAQVDYLRVLLEAKRRQPADDVYSGLVQAADESGQLSEAELVSMAHLLMMSGFETTMNMIGNALVTLLVNPQQLTLLRAQPELLPNAMEELVRHDSPVRASMLRFTVEDVELDGVTIPAGEYILVSNLTANHDAERFDDPDRLDLTRNTDGHLGYGFGVHYCVGASLARLEGRIAIQRLLARFPDLQLAVPHAELQWLPITFLRALISVPVRTGCSAPANTASHANPIERIAQ

Gene 4609 [putative cytochrome P450 hydroxylase]

atggacgacgcattcagcgaggaaggcagcgcgcagccgcgccatgacgctcagcgtccggcgctcgccccgcggagcgacggtttcgacatacacacctaccatcccgacttcgtcgccgatccctatccgctgttgcggctgatccgctcccgtgcgccggtctgccgcgaccaggcctcgatctggtggatcagtcgctatgccgatgtttcggcgtgcctgcgcgaccgccgtttctccgccgaccccgctcgccttggcgccgccggcgtccgccagggcggcgccagctggttcggccaccagcaattgcaaccgctggcgcgcttctacgacaacttcatgctgttcaacgacgcgccgcggcacacccgcctgcgcaggctgttcgctccggccttcggccccgacgccgtgcgccgctgggaagcgcgcatcgaggtgctggtggaagaactcctcgacagcttgctggagcgccgcgagcccgatctgctcagggatttcgccgaaccgctgacgatccgggtggccgccgagctgttcggctttccccgcgaagataccgggcaactgctgccctggggccgcgatctggccgccgggctcgatctcgccgccagccacggcgatgccgggcagatcaaccgcagcgcggccgccttcagcgactacctgcaacgccaggcgcgcggctggagcgatggctcttcgcgcccgccgtccggtgcggcgccgagcatcctcgacggcgccgcgatgctggaggccggactgggcctcgaggacctggtagcggcctatgccatggtgttcatggccgccttcgaaaccaccatcagcatggtcggcaacgctacgctggcgctgctcacccatcccgaccagctcgacctgctgcggcggtgcccggagttggcggcgaacgcggtggaggaactgctgcgcttcgatggcgcggtgcgcggcggcgtgcgttgcaccctggaggaggtggagatcggcggccagcggattccgcctggagagaaggtctggctgagcttcctcgcggcgaaccgcgacccggagatgttcgccgcccccgaccgcctgcagttgcagcgagcgaacgccaagcagcacgtggcgttcgcccatggtccgcactactgcctgggcgcgtacctggcccggctcgaattgcaatgcgcattgcgcggcctggtgcgtcgccgtttcgccctggcctcggaaccgacggacctgcgctggcggcgcagctcggtgttccgcaccctggagcgcttgccgatcgtcccggaaggcgacgctcagaagacctgcgagtag

MDDAFSEEGSAQPRHDAQRPALAPRSDGFDIHTYHPDFVADPYPLLRLIRSRAPVCRDQASIWWISRYADVSACLRDRRFSADPARLGAAGVRQGGASWFGHQQLQPLARFYDNFMLFNDAPRHTRLRRLFAPAFGPDAVRRWEARIEVLVEELLDSLLERREPDLLRDFAEPLTIRVAAELFGFPREDTGQLLPWGRDLAAGLDLAASHGDAGQINRSAAAFSDYLQRQARGWSDGSSRPPSGAAPSILDGAAMLEAGLGLEDLVAAYAMVFMAAFETTISMVGNATLALLTHPDQLDLLRRCPELAANAVEELLRFDGAVRGGVRCTLEEVEIGGQRIPPGEKVWLSFLAANRDPEMFAAPDRLQLQRANAKQHVAFAHGPHYCLGAYLARLELQCALRGLVRRRFALASEPTDLRWRRSSVFRTLERLPIVPEGDAQKTCE

Gene 3206 [monooxygenase, flavin-binding family]

atgcctgtcgaacacctggacgtactcatcgtcggcgccggattgtccggcgtcggcgccgcctaccacctgatgaagcattgccccggcaagagcttcgccctgctcgaagggcgcgcggcgatgggcggtacctgggacctgttccgctaccccggcatccgttccgactcggacatgttcaccctcggctacaacttcaagccatggagcgatcccaaggccatcgccgacggtccgtcgatccgccgctacatcgaggagaccgcacgggagaacggcatcgaccggaagatccgctaccgccaccgggtgctcagggccgactgggattccgccaatgcgcgctggaacctcgacgtgcagcgcggcgacgagcccgagccgctgcgcatgaccgcgcagttcctgctgatgtgcaccggctactaccgctacgaggccggctataccccggaattcgtcggtcgcgaggacttcgccgggcaggtcgtccatccgcagctgtggcccgaggacctcgactacagcggcaagaaggtggtggtgatcggtagcggcgcgaccgccgtgaccctggtgccgtcgctgaccgacaaggccgcccacgtcaccatgctgcagcgctcgccatcctacgtgatcaccctgccgcagaaggacgcgatctccaatttcctccgccgcttccttccggaaacctggatctaccgccaggcgcgggcgcgcaacgtggccatgcagatggtcttcttcatgctcgccaggaccttcccctgcctggtccgcaaggccctgctcaagctggccagcctgcaactgggcaaacgcttcgacatgcgtcacttcagcccgcgctacaagccgtgggacgagcgggtctgcgcggtgccggacggcgacctgttcaaggtgctgcgcaagggcaaggcgtcggtggtcaccgagcacatcgaccgcttcgtcgagcggggcattcgcctgaagaccggcgaggtgctggaggcggacatcatcgtcaccgccaccggcctcgacctggtgatgttcggcggcgccgagctggcggtcgacggcaagccgttccaggtcaaccagagcatgggctatcgcggcatcatgctgcgcgacctgccaaacctggcggcggtggtcggctataccaacgccagttggacgctcaaggcggacctctccagcgaatacttctgccgcctgatcaatcacatggatgccaccggcatgcgccaggtgaccgcccgcgacagcaccggcgacgtgcgcgaggagcccttcctcaacctcgactccggctacatccagcgcgccgccgagcgcatgcccaagcagggcgaccggatgccctggaagctttaccagaactacgtcctcgacctggcgctgctgcgctacggcaaggtcgaggacggctacctggtgttctcctcgccggcgccgcagcggcaagccggcggcgcggcggtgcaggccctgggctga

MPVEHLDVLIVGAGLSGVGAAYHLMKHCPGKSFALLEGRAAMGGTWDLFRYPGIRSDSDMFTLGYNFKPWSDPKAIADGPSIRRYIEETARENGIDRKIRYRHRVLRADWDSANARWNLDVQRGDEPEPLRMTAQFLLMCTGYYRYEAGYTPEFVGREDFAGQVVHPQLWPEDLDYSGKKVVVIGSGATAVTLVPSLTDKAAHVTMLQRSPSYVITLPQKDAISNFLRRFLPETWIYRQARARNVAMQMVFFMLARTFPCLVRKALLKLASLQLGKRFDMRHFSPRYKPWDERVCAVPDGDLFKVLRKGKASVVTEHIDRFVERGIRLKTGEVLEADIIVTATGLDLVMFGGAELAVDGKPFQVNQSMGYRGIMLRDLPNLAAVVGYTNASWTLKADLSSEYFCRLINHMDATGMRQVTARDSTGDVREEPFLNLDSGYIQRAAERMPKQGDRMPWKLYQNYVLDLALLRYGKVEDGYLVFSSPAPQRQAGGAAVQALG
